# A System for Heart Sounds Classification

**DOI:** 10.1371/journal.pone.0112673

**Published:** 2014-11-13

**Authors:** Grzegorz Redlarski, Dawid Gradolewski, Aleksander Palkowski

**Affiliations:** Department of Mechatronics and High Voltage Engineering, Gdansk University of Technology, Gdansk, Poland; University of Minnesota, United States of America

## Abstract

The future of quick and efficient disease diagnosis lays in the development of reliable non-invasive methods. As for the cardiac diseases – one of the major causes of death around the globe – a concept of an electronic stethoscope equipped with an automatic heart tone identification system appears to be the best solution. Thanks to the advancement in technology, the quality of phonocardiography signals is no longer an issue. However, appropriate algorithms for auto-diagnosis systems of heart diseases that could be capable of distinguishing most of known pathological states have not been yet developed. The main issue is non-stationary character of phonocardiography signals as well as a wide range of distinguishable pathological heart sounds. In this paper a new heart sound classification technique, which might find use in medical diagnostic systems, is presented. It is shown that by combining Linear Predictive Coding coefficients, used for future extraction, with a classifier built upon combining Support Vector Machine and Modified Cuckoo Search algorithm, an improvement in performance of the diagnostic system, in terms of accuracy, complexity and range of distinguishable heart sounds, can be made. The developed system achieved accuracy above 93% for all considered cases including simultaneous identification of twelve different heart sound classes. The respective system is compared with four different major classification methods, proving its reliability.

## Introduction

Cardiovascular diseases are the single leading cause of death worldwide. An estimated 17.3 million people died in 2008 and the number is projected to reach 23.3 million by 2030 [1]. Every year a million heart cases and over one hundred thousand of myocardial infarctions occur in Poland only. Regular heart tests may allow to detect heartbeat irregularities and help to avoid heart complications, greatly increasing the chance of recovery. Because of a fast life pace, the development of non-invasive auto-diagnostic systems, that would allow to carry out a preliminary medical examination at home without doctor participation, becomes the subject of research for many scientists.

One of the methods that meets all the standards is phonocardiography (PCG), defined as monitoring of the human circulatory system by registering biomechanical activity of the heart. Despite its simplicity and ease of implementation, it is rarely used for cardiac diagnosis mainly due to the lack of reasonable solutions, that could allow to unambiguously interpret the results. On the other hand, this technique belongs to a group of methods whose development is particularly needed in self-analysis systems (such as smart stethoscopes). The need to develop efficient methods for self-diagnosis is emphasized in the context of long and lonely expeditions, such as marine, polar, space, etc. This kind of solution would allow one for an early detection of pathological health states and commencement of appropriate life saving actions.

Current research in this field is focused on the development of suitable algorithms, which in the future may lead to development of an intelligent stethoscope. Because of the nature of PCG signals and undesired noise during examination, it is important to divide the diagnosis process into two steps. The first being the processing of original signals aimed to extract features, which would help to distinguish all of the types of signals, and the second associated with the process of signal classification. There were several successful attempts to develop such systems [2]–[15], where the majority of works were focused on the application of techniques based on Artificial Neural Networks (ANN) and Support Vector Machines (SVM).

One of the earliest heart valve disease detection systems based on ANN was developed by Turkoglu, Arslan and Ilkay [14], who used wavelet entropy and short-time Fourier transform to determine specific features of heart signals, consequently obtaining classification accuracy of 94% for normal heart sounds, and 95.9% for pathological ones. Wavelet analysis of the PCG signal in combination with homomorphic filtering and K-means clustering method was presented by Gupta et al. (leading to 97% accuracy in distinguishing two abnormal and one normal heart states) [13]. A multilevel wavelet decomposition with a multilayer perceptron trained by a back-propagation algorithm achieved 94.42% of accuracy in identifying four heart states [10]. Other works include the use of multivariate matching pursuit to model murmurs and classifying them with a three-layer feed-forward perceptron network with 92.5% of accuracy (distinguishing normal from abnormal heart states) [11] or a combination of detection of characteristic heart features (activity, complexity, mobility and spectral peaks) with ANN, providing a rate of 98% in identification, however able to distinguish only three of them [8].

Another group of methods employ the SVM as the main classifier. An approach for heart sounds identification presented by Wu et al. ensured 95% of accuracy using wavelet transform to extract the envelope of PCG signals [4]. However, the authors were able to distinguish only normal from abnormal heart states. The same results were achieved by Jiang and Choi [5] who developed a system for in-home use, however, this system was proven only by a case study. A diagnosis system based on principle component analysis connected with an adaptive network was developed by Avci and Turkoglu [6]. In this case the system ensured 96% accuracy in classification of normal and 93.1% of two abnormal heart states. Later, Avci improved this system and developed genetic Support Vector Machines, which gave 95% of accuracy [7]. There are also examples of using wavelet transforms and short time Fourier transform methods for feature extraction [3] or using wavelet decomposition to distinguish two out of five types of heart states with up to 93.42% accuracy [15].

Other notable works introduced solutions such as: an analytical model, based on a single-DOF for extracting characteristic waveforms from cardiac sounds and a fuzzy C-means clustering method for their classification [5]; the use of pre-defined function blocks associated with different physical phenomena and identifying heart states on this basis [12]; time-frequency analysis in conjunction with Rényi entropy [9] or Mel-frequency cepstral coefficients with hidden Markov models [2].

Since the quality of phonocardiography signals is no longer an issue, the development of appropriate algorithms, capable of distinguishing a great number of heart diseases based on relevant heart sounds, becomes an essential issue. The main problems connected with the development of relevant techniques are the wide variety of distinguishable pathological heart sounds and non-stationary character of PCG signals. Bearing in mind those issues, a question is raised how to increase the variety of distinguishable heart sounds and improve performance of such systems in terms of reduction of their computational complexity without compromising the precision. Therefore, a new system for heart sounds identification is proposed. The system consists of a feature extraction part carried out by estimating Linear Predictive Coding (LPC) parameters and signal classification performed by Support Vector Machine with a Modified Cuckoo Search (MCS) optimizer.

The main objective of this paper is the development of a system capable of simultaneous recognition of many pathological heart sounds with good accuracy. The system is validated by twelve different sets of waveforms representing various normal and abnormal heart sounds. The performance of the SVM-MCS classifier is compared with a classifier based on Artificial Neural Network and three types of SVM classifiers with different kernel functions.

## Methods

### Problem Formulation

The described problem is twofold. The PCG signal must be properly processed and all characteristic features extracted in order to provide sufficient information for classifiers. Most of the related works on this matter [3], [4], [10], [13]–[15] employed several types of wavelet analysis, which is a computationally complex technique. In this paper it is shown that by using spectral analysis (used in the LPC algorithm) it is possible to provide sufficient information for the classifier and maintain little computation complexity. However, with this method a new problem rises up – a large number of parameters and associated large search space.

The problem of PCG signals classification is non-trivial. Minor differences between the characteristics of particular abnormal PCG signals and a large feature space (in the case of using, e.g., LPC coefficients) make the task unsuitable for many methods [16]. The classification problem can be described as follows. Giving a set of input values 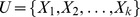, where 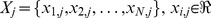, the task is to find hyperplanes dividing the *N*-dimensional search space in order to enable the separation of *m* distinguishable classes *S* [16]. A hyperplane can be denoted as
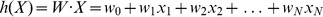
(1)where *x* and *w* are a part of the augmented feature and weights vectors, respectively.

As shown in [15], amongst all mentioned methods, those based on a Support Vector Machine classifier are characterized by best performance. Moreover, based on the literature [17] and results of classification for individual heart states presented in Table 1, it can be stated that choosing a particular one kernel function and a set of parameters may not produce sufficiently accurate results for all the cases, thus a dynamic model of kernel function and parameters selection are necessary.

**Table 1:** Percentage of correctly classified signals for different heart sounds.

Heart state	ANN	SVM-poly	SVM-rbf	SVM-quad	SVM-MCS-ca	SVM-MCS-sv
Ejection click	92.56	85.99	83.15	79.42	94.40	89.56
Normal split S1	73.67	83.17	83.15	83.94	95.60	90.91
Normal split S2	55.33	80.52	81.26	78.73	91.46	89.01
Late systolic murmur	96.22	88.83	87.25	88.69	97.31	92.87
S1	95.75	88.30	81.64	88.38	94.63	92.62
S2	75.71	91.01	81.64	91.26	97.25	94.44
S3	100.00	83.88	81.64	83.23	96.63	91.85
S4	46.20	91.26	83.15	95.58	96.66	96.22
Pansystolic murmur	91.50	81.71	83.15	82.85	96.15	88.70
Early systolic murmur	100.00	85.82	82.40	85.04	93.65	90.69
Opening snap	83.00	82.24	81.26	82.40	93.90	88.29
Diastolic rumble	66.00	85.57	81.99	85.76	97.53	92.08
**Average**	81.33	85.69	82.64	85.44	95.43	91.44
**Average var.**	301.34	90.15	99.46	106.38	17.50	35.17

ANN – Artificial Neural Network, SVM-poly – Support Vector Machine with polynomial kernel function, SVM-rbf – Support Vector Machine with radial basis kernel function, SVM-quad – Support Vector Machine with quadratic kernel function, SVM-MCS-ca – Support Vector Machine with Modified Cuckoo Search optimizer and classification accuracy fitness function, SVM-MCS-sv – Support Vector Machine with Modified Cuckoo Search optimizer and support vector number fitness function, var. – variance

**Figure 1:**
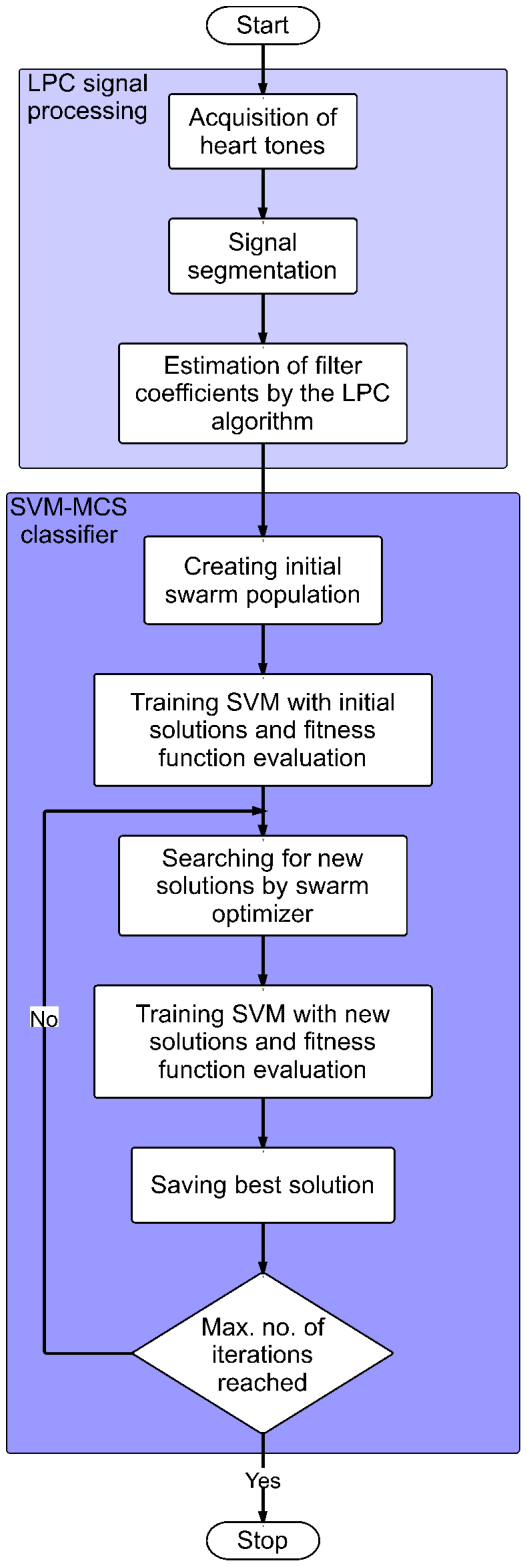
A flowchart of the hybrid LPC-SVM-MCS system training process. The system first collects PCG signals and performs their segmentation to extract useful information for LPC estimation. Then the training process commences where the Modified Cuckoo Search algorithm optimizes parameters of a Support Vector Machine classifier.

The presented system is divided into two parts, as shown in Figure 1. In the first part, a modified LPC algorithm [18] is introduced. Its aim is to solve non-stationary problem of phonocardiographic signals, as well as enable to increase the number of distinguishable states. Its operation forms a basis for a Support Vector Machine-Modified Cuckoo Search classifier (SVM-MCS), responsible for identification of the signals.

### Modified Linear Predictive Coding Algorithm

The LPC algorithm is widely used in speech signal processing. Fundamentally, the algorithm is based on matching the human vocal tract as a modelled filter in 20–30 ms quasi-stationary time intervals. The first attempt to adapt the LPC algorithm to simulate heart sounds was made by Agostinho and Souza [19]. In order to minimize the difference between original and simulated signals, the authors decided to use a fixed 30 ms rectangular window frame, as well as to increase the number of impulses in excitation function, and to set the fifth order of the filter. However, those changes made it difficult to classify the signals based on the determined coefficients of the filter. In order to design an effective classifier it is necessary to introduce certain changes to the process of modelling PCG signals by the LPC algorithm.

**Figure 2:**
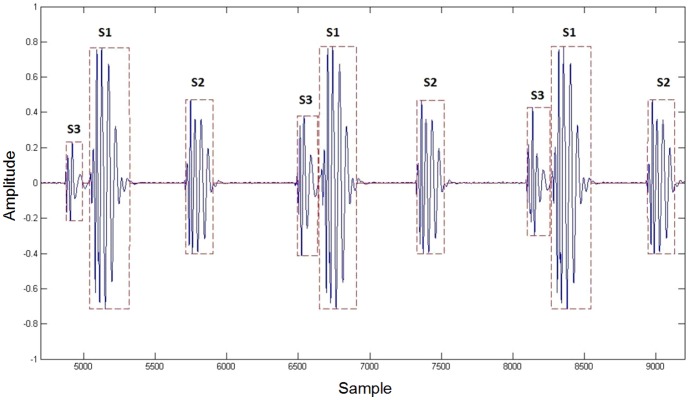
Result of the heart tone segmentation algorithm. The waveform presented contains normal S1, S2 and S3 heart sounds, which are segmented by a variable size time window for further analysis.

Heart sounds are characterized by a large variation in both time and frequency domains. Therefore, the PCG signals – like most biological signals – are classified as non-stationary. Thus, it is strictly inadvisable to divide PCG signals using a fixed size time window, as it is used in the case of speech signal coding. For this reason a special algorithm was developed that separates each heart tone using a variable size time window. Figure 2 presents the result of the separation task. Each of these frames determine the parameters of the filter.

**Figure 3:**
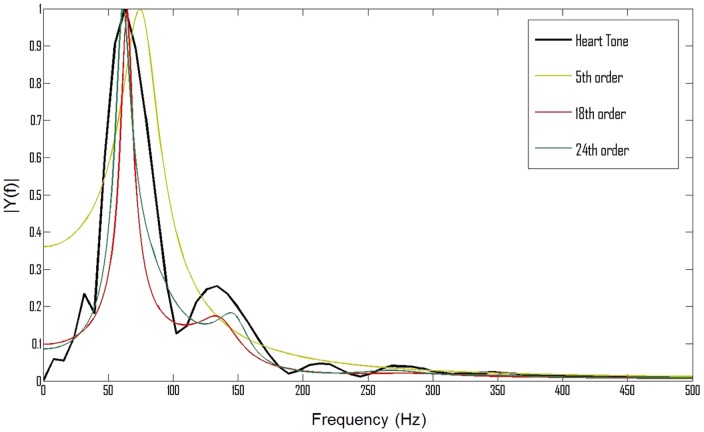
A S3 heart tone and LPC filter spectra. A comparison between a real S3 heart tone spectrum and spectra of filters estimated by the LPC algorithm. The 24th order filter provides the closest representation of the original heart tone spectrum.

Due to differences in dynamics of speech and heart sounds, in order to properly identify the recorded signals, it is necessary to change the order of the filter describing the signal spectrum. Assuming that there could be additional peaks in the case of cardiac pathologies, in order to map the spectrum at least nine transmittance poles are needed. To ensure real coefficients of the polynomial, it is necessary to couple the poles in pairs, which requires at least an 18th order transmittance [18]. Figure 3 presents that by the use of a 24th order transmittance even a better spectrum matching is reached. Finally, the transfer function of the filter may be written as:
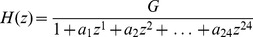
(2)


**Figure 4:**
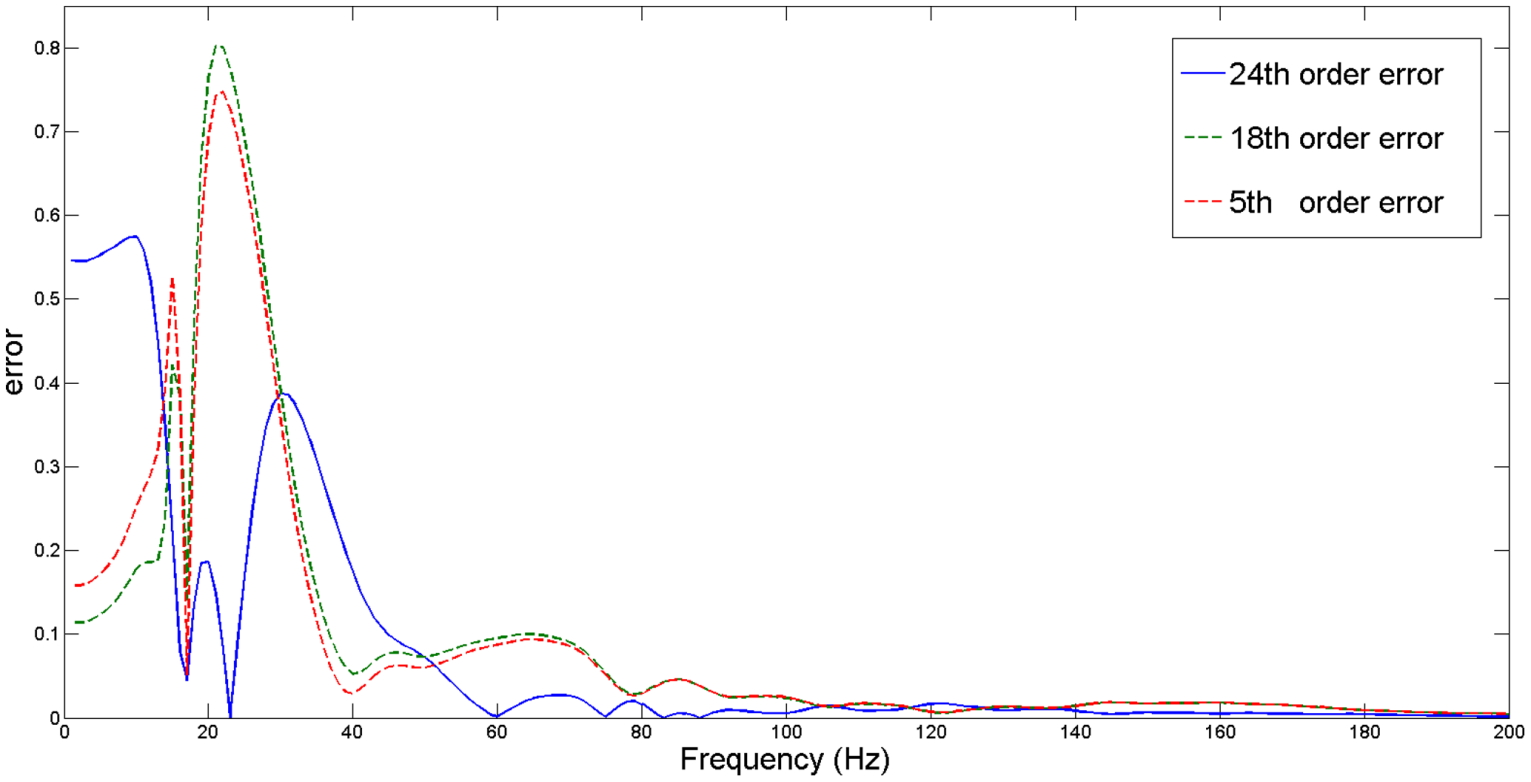
Spectrum matching error for different filter orders. A comparison of matching errors in replicating a S3 heart tone spectrum. The presented curves indicate errors for three filters estimated by a Linear Predictive Coding algorithm with a transfer function of the 5th (red dotted line), 18th (green dotted line) and 24th (blue line) order. The 24th order filter obtained significantly lower error.

To better illustrate the matching of individual filters, a comparison of their matching errors is shown in Figure 4. The error indicates the difference between a real PCG signal spectrum and a spectrum obtained from the estimated filter. It can be observed that the 5th and 18th order filters have a significantly higher error than the 24th order filter, which is especially visible at low frequencies. Above 100 Hz all filters have a negligible matching error. Additionally, in order to emphasize that the 24th order filter is better, a fitness factor for the above mentioned filters was calculated according to the following formula:
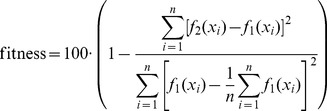
(3)


where 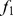 is the spectrum of the filter, 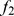 is the original PCG signal spectrum and 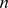 is the number of samples. According to the fitness factor, the 5th order filter obtained a 85.5% match, which indicates that the signal is not properly replicated. The 18th order filter obtained a satisfying result of 97.36%, however, even better matching was achieved by the 24th order filter, which obtained 99.7%.

**Figure 5:**
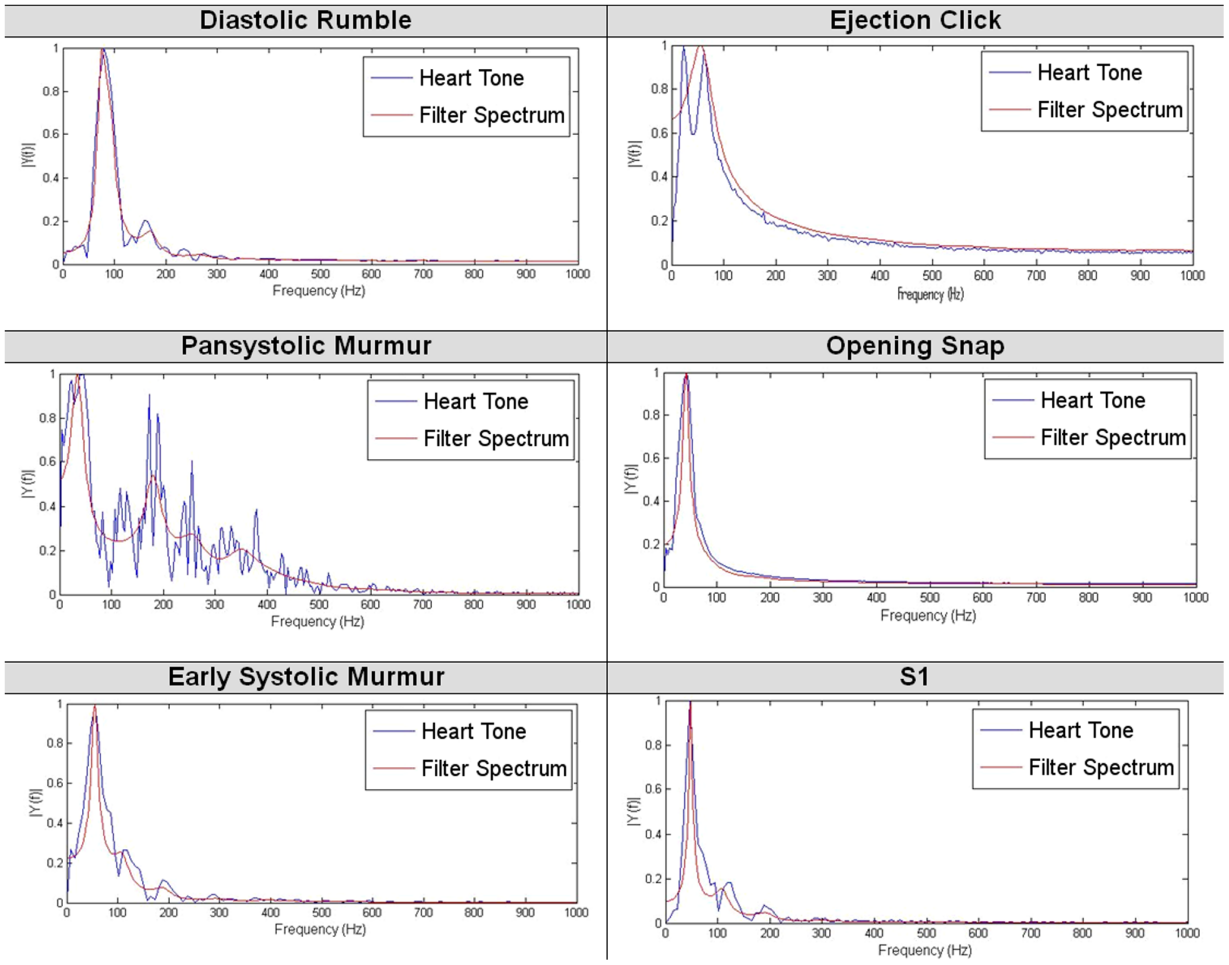
Spectrum comparison of selected heart sounds and LPC filters. Presented curves demonstrate the effectiveness of the modified LPC algorithm in estimating different heart sounds.

It can be observed that the spectrum of the 5th order filter (determined by Agostinho and Souza [19]) does not follow the spectrum of the original signal. Therefore it is impossible to build an effective classifier based on the parameters of the fifth order filter. As expected, the spectrum of the eighteenth order filter provides much better accuracy in matching the real signal. However, in this case a small shift between the original and modelled spectra occurs. Therefore, it was decided to use the 24th order of the transmittance function, which is capable of eliminating this drawback. By further increasing the order of the filter, further improvement of the spectra matching can be achieved. However, the feature space increases as well. As it is shown in the next part of this paper, the 24th order filter provides sufficient information for the classification task. In order to demonstrate the effectiveness of the resulting changes, a spectrum comparison of selected heart sounds is presented in Figure 5.

As seen in Figure 5, each heart tone spectrum has its characteristic shape, which is properly followed by the spectrum of the selected filter. Consequently, based on the parameters of the filter (which were used to determine the shape of the spectrum), it is possible to distinguish heart sounds. The 24 coefficients (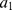
–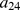) from the denominator of the transfer function of the filter (Equation 2) were used as signal inputs for the SVM-MCS classifier, which is described throughout the next sections.

### Support Vector Machine Classifier

Support Vector Machine is a supervised machine learning technique introduced by Vapnik et al. [20], [21]. Its aim is to solve a binary classification problem by designation of an optimal hyperplane (Equation 1) separating two classes, labelled as 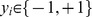. The hyperplane meets the requirement of having a maximum margin, i.e. being maximally distant from both classes.

The SVM classifier training process is as follows. Giving a set of labelled training samples 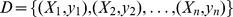, belonging to two classes, the SVM produces a linear decision boundary separating the data. In the case of non-linear decision surfaces, the process comes down to solving the following optimisation problem [22]:
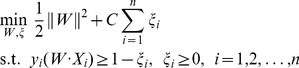
(4)where 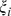 are positive slack variables and *C* stands for a penalty parameter.

In order to cope with non-linearity of the feature space, particular kernel functions are used. The most common functions and their parameters (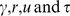) are polynomial functions (5) and radial basis functions (6).
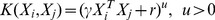
(5)

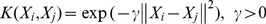
(6)


The described classification problem requires more than two classes to be handled. There are three strategies for dealing with multi-class classification problems with SVM: one-against-one, one-against-others and all-together [17]. In the one-against-one strategy the result of classification is obtained by comparison of pairs of data sets which belong to different classes, giving 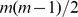 classifiers. The one-against-others separates each class from the rest and creates *m* classifiers. The last strategy solves one single optimisation problem for all considered classes. In the presented work, the one-against-others strategy was chosen because it led to the best results in initial tests and provided less computation complexity in comparison with the one-against-one strategy (less support vector classifiers produced).

When developing a SVM classifier, one must choose values for the parameters of the support vectors. Those parameters have huge impact on training efficiency of the classifier and strongly depend on the classification problem itself. While those parameters cannot be chosen randomly, it is essential to optimise their values for a given problem. Therefore an additional optimisation system is needed.

### Modified Cuckoo Search Optimizer

The problem of creating an efficient and flexible classifier based on the Support Vector Machine requires the use of an optimisation method for tuning particular classifiers. Presently, increased attention is attracted by optimisation techniques based on swarm intelligence. The swarm intelligence is a new field of artificial intelligence dealing with the design of multi-agent systems for optimisation applications. The inspiration behind creating swarm intelligence methods was the behaviour of groups of social insects, such as ants or bees, thus its assumptions are fundamentally different from the traditional approach for solving optimisation problems. Instead of using a single, complicated apparatus of master control over the entire optimisation process, swarm intelligence is based on the cooperation of many simple units that affect the whole operation. There is a kind of interaction, known as emergence, where relatively simple actions of individual agents and their interaction leads to the formation of an emergent behaviour.

Swarm algorithms are a set of stochastic metaheuristics that are widely used in various optimisation tasks [23]. It has been proven that a swarm algorithm itself is an efficient technique on which a classifier can be build [16]. Moreover, there are many works where the application of a swarm algorithm for optimisation of a SVM gave great results and proven to be better than other methods [22], [24], [25].

One of the newest swarm algorithms is the Cuckoo Search, developed by Yang and Deb [26]. It was inspired by the brood parasitism phenomenon seen in some species of cuckoos, which place their eggs in nests of birds of different species. The algorithm applies the mechanism of Lévy flights to select subsequent nests, allowing for proper balance between exploration and exploitation of a search space. The main assumptions of the algorithm are:

- each cuckoo lays one or more eggs (in a randomly chosen nest) representing the coordinates of a point in the search space, being the problem solution,
- some nests with the best value of fitness function are moved to the next iteration,
- the number of nests is fixed and at the end of each iteration a part of them is rejected with some probability.

The literature shows that it is more efficient than other swarm algorithms [23], [26], [27]. Furthermore, there are documented examples of applying this algorithm to a classification task with success [28]–[30].

The Modified Cuckoo Search [31] is a promising modification of the original algorithm. In comparison to the original algorithm, MCS differs mainly by using the golden mean method in the strategy of creating new nests, by using a variable length of the cuckoo flight, as well as it introduces a more greedy policy of promoting best solutions. Due to its better efficiency than the Cuckoo Search [31], use of the Modified Cuckoo Search algorithm for the relevant problem is proposed.

In order to deal with the issues related to the process of classifying LPC-modified PCG signals with a SVM, the following modifications to the optimisation process are introduced. One of the problems is a large feature space of the LPC filter parameters where not all of them represent necessary information for the classifiers. Therefore, the output of the MCS algorithm includes 24 binary variables indicating which parameters are used for the training process. Moreover, to provide a dynamic model of the kernel function and parameters selection, the output also indicates which one of the common kernel functions (linear, quadratic, polynomial or radial basis function), as well as the values of their respective parameters (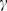 or *u*) and the values of the penalty parameter *C*, are selected for each one of the SVM classifiers working with the one-against-others strategy. Therefore, the dimensionality of the solution search space is 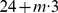, while the space contains both real and binary variables.

Another important aspect is the objective function selection. There are several types of functions which can be used for optimising SVM classification [17], one of them being the support vector count or the performance of the classifier. In the case of imbalanced datasets, an useful performance measure is the balanced accuracy (7) which avoids inflated performance estimates. It is defined as the arithmetic mean of sensitivity and specificity, which are calculated by knowing the *m* binary outputs of the classifiers (indicating membership to given classes). Overall performance is calculated by conducting a leave-one-out test for all training samples.
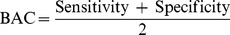
(7)where
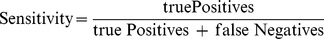
(8)

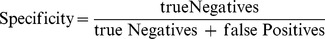
(9)


**Figure 6:**
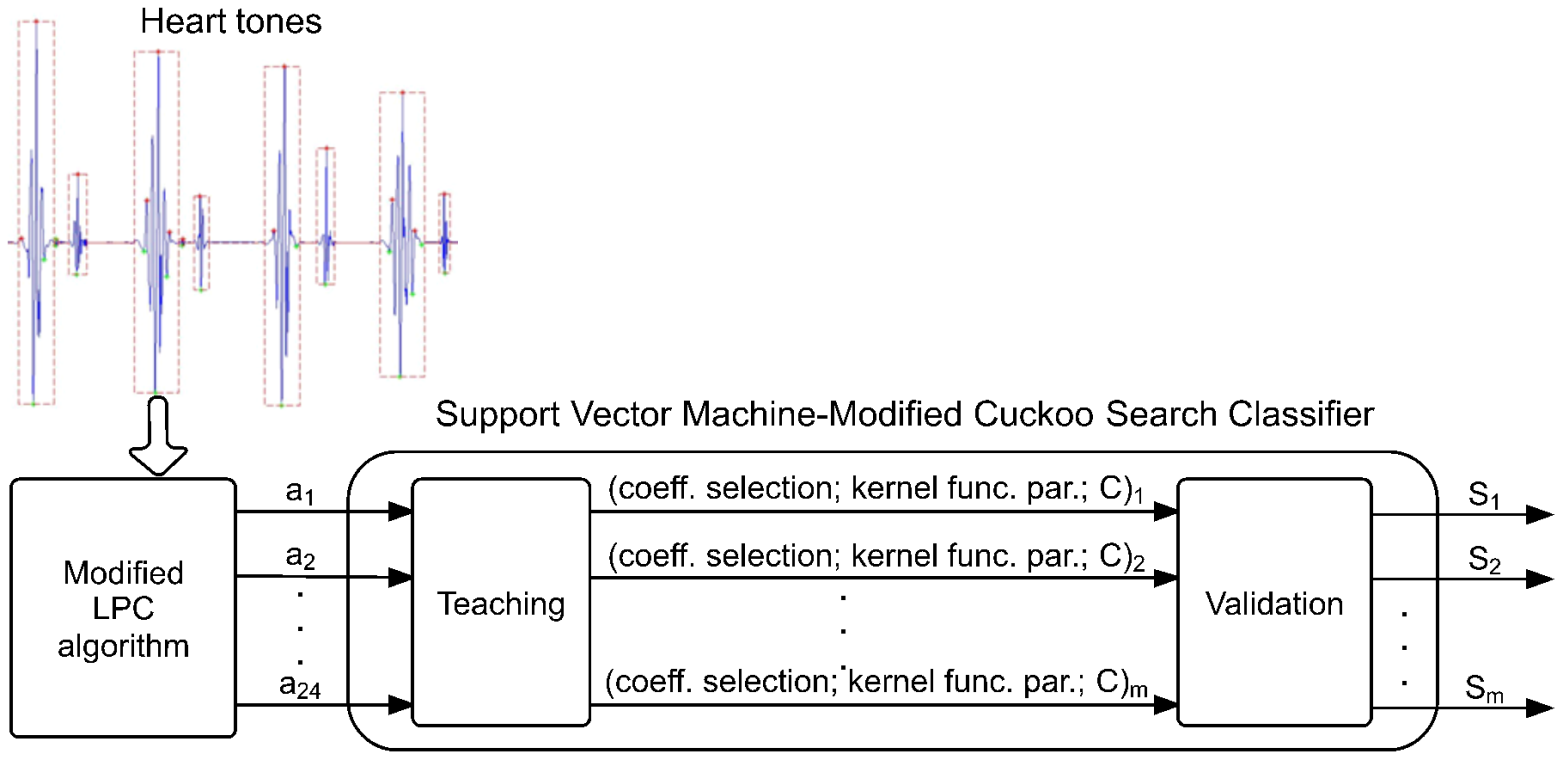
Data flow in the proposed classification system. The picture presents data transfer in the proposed classification system. The modified LPC algorithm estimates filter coefficients 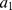
–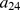 and passes them to the training part. After the training and optimisation process, the selection of appropriate coefficients, selected kernel function and its parameters, and the penalty parameter *C* can be used in validation of the testing data set.

True positives and true negatives represent the number of correctly classified samples belonging to the class being tested and other classes, respectively. False positives and false negatives indicate the number or misclassified samples of the relevant classes. Finally to summarize the work of the proposed system, an overview of data flow is shown in Figure 6.

## Results and Discussion

**Figure 7:**
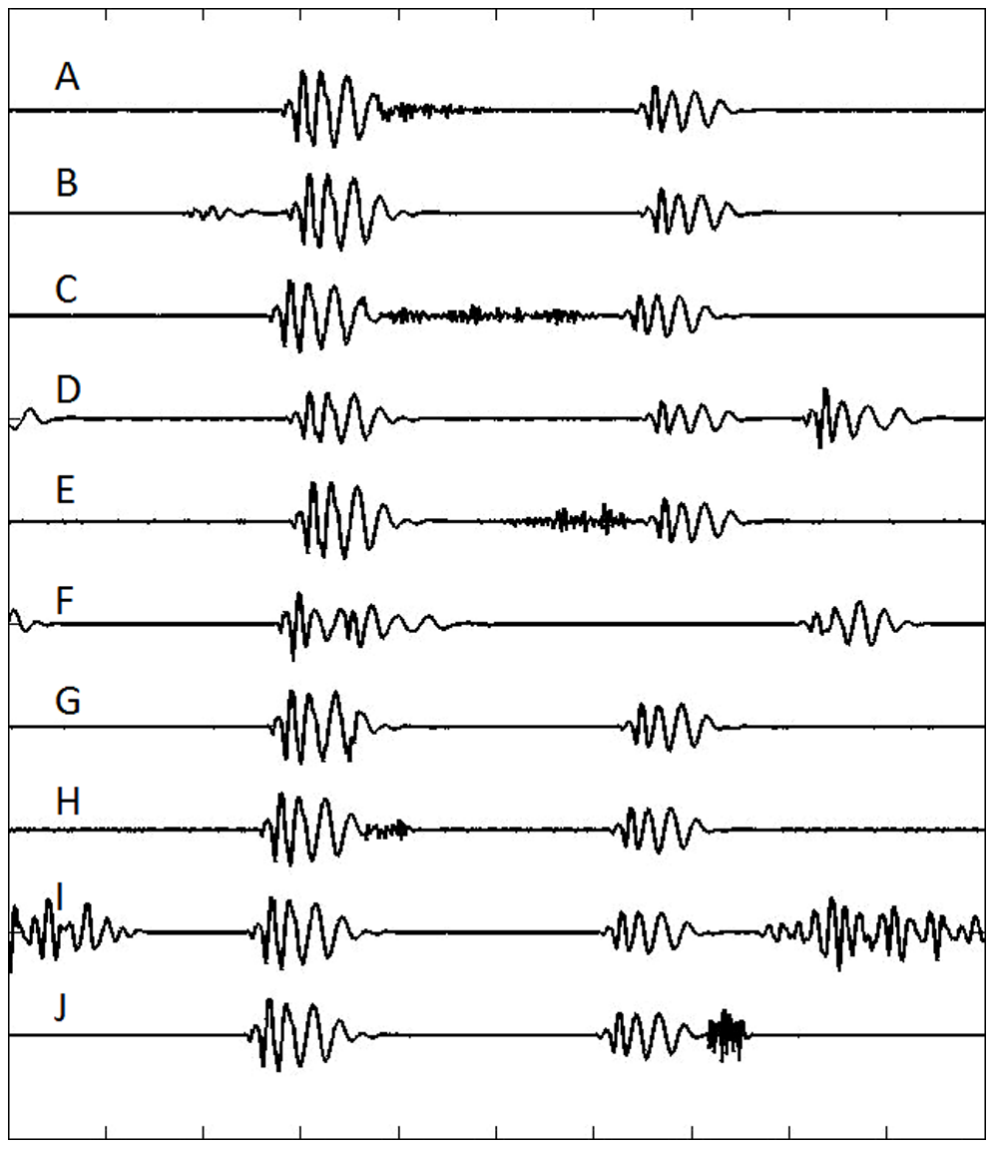
Example heart sounds used in the tests. A – early systolic murmur, B – S4, C – pansystolic murmur, D – S3, E – late systolic murmur, F – normal split S2, G – normal split S1, H – ejection click, I – diastolic rumble, J – opening snap.

The proposed system was implemented in Matlab. The LPC algorithm was used to create a database containing eight types of pathological heart sounds (early systolic murmur, pansystolic murmur, late systolic murmur, normal split S2, normal split S1, ejection click, diastolic rumble and opening snap) and four normal heart sounds (S1, S2, S3, S4). The considered waveforms of heart sounds were taken from a public database [32] and their examples are presented in Figure 7. The database consisted of six waveforms of each type, all originating from an unknown human source. Initial test were carried out by dividing in half all samples to create a set of training and testing samples (Table 1). However, due to the fact that only seventy two PCG samples were available, a leave-one-out testing strategy was also adopted to improve statistical significance of the results (Table 2).

**Table 2:** Percentage of correctly classified signals for different heart sounds from a leave-one-out test.

Heart sound	ANN	SVM-poly	SVM-rbf	SVM-quad	SVM-MCS
Ejection click	100.00	90.28	91.67	90.28	93.06
Normal split S1	83.33	91.67	91.67	90.28	93.06
Normal split S2	66.67	91.67	91.67	88.89	94.44
Late systolic murmur	100.00	94.44	94.44	93.06	93.06
Pansystolic murmur	83.33	91.67	91.67	87.50	95.83
Early systolic murmur	83.33	91.67	91.67	91.67	93.06
Opening snap	100.00	90.28	91.67	87.50	93.06
Diastolic rumble	100.00	94.44	91.67	93.06	93.06
S1	66.67	93.06	91.67	94.44	94.44
S2	100.00	93.06	91.67	94.44	97.22
S3	100.00	90.28	91.67	91.67	98.61
S4	83.33	90.28	91.67	93.06	97.22
**Average**	88.89	91.90	91.90	91.32	94.68
**Average var.**	833.33	18.97	1.29	33.92	33.44

ANN – Artificial Neural Network, SVM-poly – Support Vector Machine with polynomial kernel function, SVM-rbf – Support Vector Machine with radial basis kernel function, SVM-quad – Support Vector Machine with quadratic kernel function, SVM-MCS – Support Vector Machine with Modified Cuckoo Search optimizer, var. – variance

Apart from the SVM-MCS classifier, an Artificial Neural Network and three types of Support Vector Machine classifiers (with polynomial, quadratic and radial basis kernel functions) were created for the purpose of comparison. The structure of the ANN was chosen according to initial simulations in order to improve the results. A network with 24 neurons with adaptive learning rate backpropagation algorithm was chosen. The compared four methods were trained with all 24 LPC coefficients. The results of this comparison are presented in Tables 1, 2, 3 and 4.

**Table 3:** Classification accuracy of compared methods for various number of considered classes.

	ANN		SVM-poly		SVM-rbf		SVM-quad	
No. of classes	Acc. [%]	Var.	Acc. [%]	Var.	Acc. [%]	Var.	Acc. [%]	Var.
2	100.00	0.00	74.36	346.84	66.66	176.75	68.67	273.74
3	100.00	0.00	78.55	47.02	67.59	2.58	80.03	81.99
4	100.00	0.00	78.72	23.68	75.76	1.00	81.25	54.25
5	100.00	0.00	82.25	15.42	80.67	0.44	84.14	19.54
6	94.33	160.56	85.45	1.87	83.79	0.21	86.72	2.29
7	90.29	235.92	86.73	0.35	86.05	0.12	88.86	0.92
8	83.00	289.00	88.54	0.27	87.76	0.07	89.49	1.66
9	77.56	750.69	89.76	0.10	89.09	0.04	90.19	0.48
10	76.50	1127.85	90.67	0.15	90.22	0.02	90.92	0.54
11	72.55	1181.70	91.66	0.01	91.13	0.01	91.46	0.04
12	71.83	532.81	92.36	0.00	91.90	0.00	92.12	0.00
**Average acc.**	87.82		85.37		82.78		85.80	

ANN – Artificial Neural Network, SVM-poly – Support Vector Machine with polynomial kernel function, SVM-rbf – Support Vector Machine with radial basis kernel function, SVM-quad – Support Vector Machine with quadratic kernel function, Acc. – Accuracy, Var. – Variance

**Table 4:** Classification accuracy of proposed methods for various number of considered classes.

	SVM-MCS-ca		SVM-MCS-sv	
No. of classes	Acc. [%]	Var.	Acc. [%]	Var.
2	100.00	0.00	100.00	0.00
3	98.67	5.35	93.90	17.66
4	94.58	14.93	88.27	11.94
5	94.33	6.85	88.00	7.56
6	94.32	3.23	87.44	5.23
7	93.08	1.08	88.91	2.17
8	92.33	1.53	90.20	1.16
9	92.76	0.91	90.69	0.59
10	92.93	0.31	91.61	0.42
11	93.08	0.06	92.34	0.35
12	93.23	0.04	92.54	0.20
**Average acc.**	94.48		91.26	

SVM-MCS-ca – Support Vector Machine with Modified Cuckoo Search optimizer and classification accuracy fitness function, SVM-MCS-sv – Support Vector Machine with Modified Cuckoo Search optimizer and support vector number fitness function, Acc. – Accuracy, Var. – Variance

As can be seen in Table 1 the Support Vector Machine with the Modified Cuckoo Search optimizer and classification accuracy – according to Equation (7) – fitness function have the best average accuracy (95.43%). By comparing it with the SVM classifier with support vector count fitness function it can be stated that the SVM-MCS-ca classifier offers greater performance in terms of classification accuracy, therefore it should be further used as the main classification system. The SVM-MCS classifier with classification accuracy fitness function outperforms all other SVM-based classifiers for all considered heart sound classes, which indicates that the additional optimisation of support vector parameters is indeed necessary and benefits the classification process. Even though some heart sounds were classified better by the ANN, its average accuracy is significantly lower than the proposed method. Some of the classes were classified with a 100% accuracy by the ANN, however the accuracy of classification for other classes is low enough to reject this solution as a main heart sound classifier. This statement is supported by the average variance of the presented solutions. The SVM-MCS classifier is characterised by low variance in given results, thus being more reliable. Such bad performance of the ANN classifier may indicate that it needs a greater number of training samples to be well trained. Moreover, it is worth to notice that in the case of the ANN classifier a complete distinction between pathological and healthy heart sounds was possible. All errors were caused by misclassification of a particular type of pathology or a singular heart sound sample. As previously noted in the literature [4], [11], [14], it has been proven that ANN is a feasible solution for basic heart sounds classification, however without additional improvements it is unable to properly distinguish all individual types of sounds (be it normal or pathological). Its use in professional medical examination at this state is therefore questionable.

Table 2 presents equivalent results, however derived from a leave-one-out test. These results confirm the above that the proposed SVM-MCS with a classification accuracy fitness function is better than the other methods. With more training samples all three SVM classifiers and the ANN classifier provide better accuracy in classification. Worse result in the case of the SVM-MCS classifier (94.68%) is due to its probabilistic nature. Given more tests and training samples, its accuracy should be higher. Lower variance of results from the SVM classifiers indicates their certainty in predicting classes membership, however their average accuracy is still worse than the proposed SVM-MCS system.

**Figure 8:**
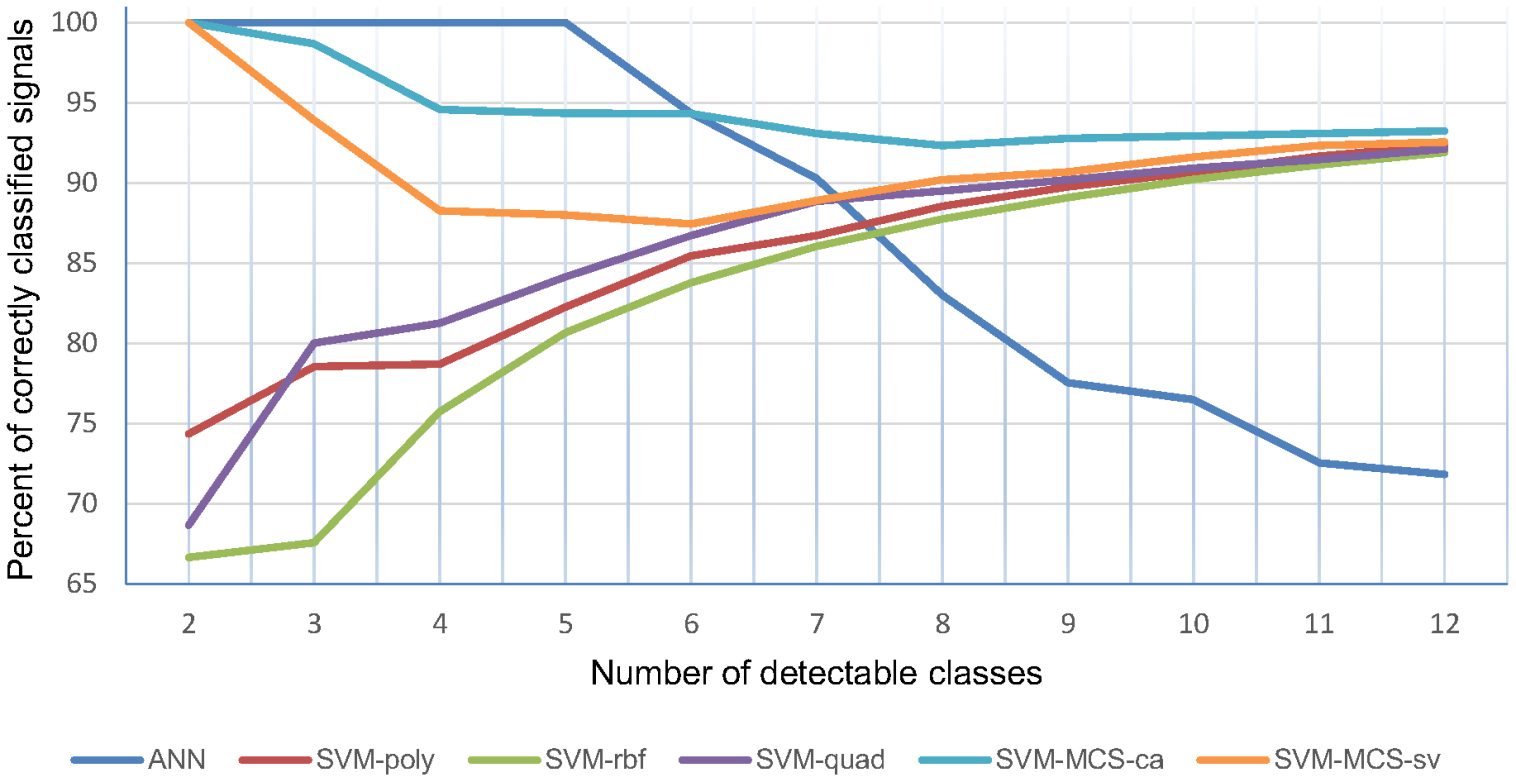
Comparison of classification results for all test groups. A comparison of accuracy of all tested methods (being: ANN – Artificial Neural Network, SVM-poly – Support Vector Machine with polynomial kernel function, SVM-rbf – Support Vector Machine with radial basis kernel function, SVM-quad – Support Vector Machine with quadratic kernel function, SVM-MCS-ca – Support Vector Machine with the Modified Cuckoo Search optimizer and classification accuracy fitness function) for a different number of recognizable classes. The SVM-MCS-ce method shows overall the best quality of classification.

Tables 3 and 4 present classification results for a different number of distinguishable classes. A graphic representation of those results can be seen in Figure 8. The Support Vector Machine with the Modified Cuckoo Search optimizer and classification efficiency fitness function classifier again has the best efficiency. The most notable result is its capability to classify 12 heart sounds at the same time with a rate of 93.23%. Its scores are never less than 92%, which compared to the other methods, presents outstanding performance. The only cases when it performs worse are the first four tests of the ANN, when the ANN achieved a perfect rate. However, for more than six classes its performance dropped significantly, which might indicate that medical examination using this classifier could be very uncertain. The performance of the SVM classifiers rises with an increase in the number of classes, reaching its maximum of 92.36% with a polynomial kernel function. It should be noted that this result is worse even when compared to the SVM-MCS system using support vector number objective function. This poor performance in the case of a small number of classes can be explained by over-fitting in the training phase caused by maladjustment of the SVM parameters.

In summary, it is notable that by providing additional optimisation, the SVM classifier outperforms other classifiers. The SVM itself is an efficient method of classification, which produces good results despite a small number of training samples, although it is susceptible to over-fitting when working with a small number of classes. Its accuracy rises further by providing more training samples, as shown in Table 2. The ANN is more sensitive to its training samples number, for which the requirements increase greatly for increased number of considered classes. By providing an additional optimisation mechanism most of the drawbacks of the SVM classifier can be diminished. In comparison to previous works, the presented system performs very well. Only few existing systems achieved better average classification accuracy [8], [13], and the reason for this was a small number of sound types taken into account.

The only disadvantage of the proposed SVM-MCS classifier is its probabilistic nature and speed of operation. While the other classifiers provided fixed results for given combination of classes and samples, the SVM-MCS classifier is more random in this manner. However, the classification accuracy of the SVM-MCS classifier is high enough to overlook this drawback. As for the speed of operation, this concerns only the training phase and is associated with a more complicated testing method due to the additional optimisation. However, after the initial training, the generalisation process of tested samples is as quick as in the other SVM classifiers.

## Limitations of the Study, Open Questions, and Future Work

The main limitation of the presented study is the number of samples, which came from only six records per type of tone. Since the number of samples is very small, the validation scenario should be considered as a case study, rather than a validation by a simulation. However, by applying the leave-one-out testing strategy, which is widely adopted in bio-system validation, it is believed that the statistical significance of the results was improved.

This system, proposed for heart sound identification, is able to classify effectively twelve different heart tone types, including various pathological and healthy sounds. Therefore, by increasing the database, carrying out studies on patients with various cardiac disease (e.g. mitral stenosis, aortic stenosis, mitral regurgitation, etc.), and by combining them with corresponding heart sounds, this system could be successfully extended to heart disease diagnosing purposes. Moreover, the combination of the LPC algorithm with the SVM-MCS classifier presents an opportunity to develop an in-home treatment system in a form of a smart stethoscope or even an implementation of the system on various mobile devices, such as smartphones or tablets.

To potentially improve the heart sound classification system, application of more recent speech coding algorithms should be tested. Cepstrum or mel-cepstrum coefficients as well as application of some time scale properties, such as variance or length of a frame, may result in increasing classification accuracy. Furthermore, performance of the algorithm in case of signals recorded in noisy environments is still an open problem. Such noisy signals may have additional picks in their frequency representation, which may introduce variance in some of the coefficients values. Moreover, development of a suitable signal segmentation algorithm is also of great importance.

## Conclusions

In this paper an attempt to find a simple artificial intelligence method for efficient heart sound classification has been successfully presented. The designed system performs well and provides satisfying results, which are generally better than those obtained from the competing methods. So far only few papers have been dedicated to the problem of classification more than four types of pathological heart sounds. The developed system correctly identified and classified eight different pathological and four normal heart sounds. What is more important, as opposed to the current state of the art, with increasing variety of distinguishable states, an increase in classification accuracy was achieved.

It has been demonstrated that by using the modified LPC algorithm it is possible to increase the amount of distinguishable states by extracting necessary information from heart sounds. In comparison with wavelet decomposition, the introduction of the LPC algorithm allows to decrease rapidly the computational complexity of the process. The Support Vector Machine-Modified Cuckoo Search classifier offers a quick, stable and efficient method of classification. It has been shown that the MCS algorithm, used as an optimizer for the SVM parameters, can reduce the feature space and select optimised values of support vector parameters to further improve the performance of the system. The main advantage of the LPC-SVM-MCS system lays in its capability to distinguish even twelve heart sounds with a particularly good accuracy of 95.43%. Also the advantage of using a classifier based on swarm intelligence and SVM is its versatility. It is known that complex decision curves can be estimated using an Artificial Neural Network, but its performance strongly depends on its structure (number of layers, number of neurons, training algorithm, etc.). Contrary to that, the SVM-MCS classifier depends only on few coefficients related to the Modified Cuckoo Search algorithm (population size, percentage of discarded eggs) so it requires no further tuning.

It is expected that future research in this area will concern, most of all, more tests on a greater number of samples. Additionally, a greater number of classes (types of heart sounds) should be introduced in order to further test the final system capabilities. In the future such system may form a base for designing a smart health monitoring device. By online measurement of heart activity it may facilitate the life of people who otherwise have to be hospitalised, or may support physicians work who otherwise will be forced to analyse a huge amount of data.
